# Expression of PD-L1 and PD-1 in Chemoradiotherapy-Naïve Esophageal and Gastric Adenocarcinoma: Relationship With Mismatch Repair Status and Survival

**DOI:** 10.3389/fonc.2019.00136

**Published:** 2019-03-13

**Authors:** Maria C. Svensson, David Borg, Cheng Zhang, Charlotta Hedner, Björn Nodin, Mathias Uhlén, Adil Mardinoglu, Karin Leandersson, Karin Jirström

**Affiliations:** ^1^Department of Clinical Sciences Lund, Oncology and Pathology, Lund University, Lund, Sweden; ^2^Science for Life Laboratory, KTH-Royal Institute of Technology, Stockholm, Sweden; ^3^Centre for Host-Microbiome Interactions, Faculty of Dentistry, Oral and Craniofacial Sciences, King's College London, London, United Kingdom; ^4^Cancer Immunology, Department of Translational Medicine, Lund University, Malmö, Sweden

**Keywords:** PD-L1, PD-1, MMR status, MSI status, esophageal cancer, gastric cancer, the cancer genome atlas

## Abstract

**Background:** The outlook for patients with esophageal and gastric (EG) cancer remains poor. Hence, there is a compelling need to identify novel treatment strategies and complementary biomarkers. Programmed death ligand 1 (PD-L1) and mismatch repair deficiency (dMMR) are putative biomarkers of response to immune-checkpoint blockade, but their prognostic value and interrelationship in EG cancer have been sparsely investigated.

**Methods:** Immunohistochemical expression of PD-L1 on tumour cells (TC) and tumour-infiltrating immune cells (TIC), and of PD-1 (programmed death receptor 1) on TIC was assessed using tissue microarrays with primary tumours and a subset of paired lymph node metastases from a consecutive, retrospective cohort of 174 patients with chemoradiotherapy-naïve EG adenocarcinoma. MMR proteins MLH1, PMS2, MSH2, and MSH6 were assessed by immunohistochemistry. The total number (intratumoural, tumour-adjacent, and stromal) of CD8^+^ T cells in each core was calculated by automated analysis.

**Results:** High PD-L1 expression on both TC and TIC, but not PD-1 expression, was significantly associated with dMMR. PD-L1 expression on TIC was significantly higher in lymph node metastases than in primary tumours. High expression of PD-L1 or PD-1 on TIC was significantly associated with a prolonged survival, the former independently of established prognostic factors. A significant stepwise positive association was found between CD8^+^ T cells and categories of PD-L1 expression on TIC.

**Conclusion:** PD-L1 expression on TIC is higher in lymph node metastases compared to primary tumours, correlates with dMMR, and is an independent factor of prolonged survival in patients with chemoradiotherapy-naïve EG adenocarcinoma. These findings suggest that PD-L1 expression on TIC may be a useful biomarker for identifying patients who may not need additional chemo- or chemoradiotherapy, and who may benefit from PD-1/PD-L1 immune-checkpoint blockade.

## Introduction

Esophageal and gastric (EG) cancers constitute a considerable proportion of cancer cases and deaths worldwide (1). The outcome of these two cancer diagnoses is in general poor, especially in Western populations with 5-year survival rates being lower than 40%. Although addition of neoadjuvant and adjuvant chemotherapy or chemoradiotherapy has led to an improved survival in patients with resectable tumours (2–7), there is still a great need to identify novel treatment strategies and complementary biomarkers. Two biological therapies that have shown efficacy for an improved overall survival (OS) in the treatment of advanced EG cancer are trastuzumab and ramucirumab, both monoclonal antibodies (mAb) targeting the human epidermal growth factor receptor 2 (HER2) and the vascular endothelial growth factor receptor 2 (VEGFR2), respectively (8–10). Immune-checkpoint blockade, e.g., targeting the programmed death receptor 1 (PD-1) pathway, is another novel upcoming treatment option that has shown promising results in various types of tumours, and the efficacy in EG cancer is being investigated in several ongoing trials. For Asian patients with heavily pretreated gastric or esophago-gastric junction cancer, the ONO-4538-12, ATTRACTION-2 study (*n* = 493) by Kang et al. has shown a significantly improved survival after treatment with nivolumab, a human IgG4 monoclonal antibody inhibitor of PD-1, compared to placebo (11). Expression of the programmed death ligand 1 (PD-L1) is a putative biomarker of response to such therapies (12), but the prognostic value in EG cancer remains unclear. PD-L1 is expressed on both tumour cells (TC) and tumour-infiltrating immune cells (TIC), whereas PD-1 is only expressed on TIC. According to a report encompassing 465 Caucasian gastric cancer cases, patients with high expression of PD-L1 on both TC and TIC had the best OS. In that study, PD-L1 was expressed on TC in 30% of the cases and on TIC in 36% of the cases. Regarding PD-1, no expression was observed on TC, whereas positive expression on TIC was denoted in 54% of the cases, and PD-1 expression on TIC was significantly associated with PD-L1 expression on both TC and TIC (13). Some studies have however reported an adverse association between PD-L1 expression and survival in gastric cancer (14, 15). In an Asian study by Zhang et al. (*n* = 132) PD-L1 expression was denoted in 51% of the gastric cancer tumours, TC and/or TIC not specified, and the 5-year survival rates was significantly better for PD-L1 positive patients. PD-1 status was not investigated in that study (15).

Mismatch repair deficiency (dMMR), or microsatellite instability (MSI), is another putative predictive biomarker of response to immune-checkpoint blockade. In the KEYNOTE-059 trial by Fuchs et al. (*n* = 259), investigating the response rate of pembrolizumab, a humanized IgG4-κ monoclonal antibody inhibitor of PD-1, in previously treated gastric and esophago-gastric junction cancer, patients with MSI-High (MSI-H) tumours had a higher objective response rate (ORR) than non-MSI-H tumours, but, notably, the majority of responders were non-MSI-H patients and only 4% of the tumours were MSI-H (12). Regarding mismatch repair (MMR) status and prognosis in gastric cancer, the reports are sparse and the results are contrasting. For example, in a study by Marrelli et al. (*n* = 472), an improved prognosis was demonstrated for patients with MSI-H gastric tumours, even tumours with more advanced nodal status, but the benefit was only confirmed in the non-cardia subgroup, with intestinal-type or tubular/poorly differentiated histology according to the WHO classification (16). On the other hand, in a report by An et al. (*n* = 1990), there was no difference in disease-free survival according to MSI status (17). Furthermore, Smyth et al. showed, in a secondary *post hoc* analysis of the Medical Research Council Adjuvant Gastric Infusional Chemotherapy (MAGIC) trial, that patients with dMMR tumours had a prolonged OS when treated with surgery alone, compared to surgery and perioperative chemotherapy together, proposing that perioperative chemotherapy may not be beneficial for patients with dMMR tumours (18). In the MAGIC trial, all dMMR tumours were found in the stomach, of note none in the lower esophagus or esophago-gastric junction. To the best of our knowledge, only one former study has investigated the relationship between MMR status and prognosis in esophageal adenocarcinoma and no significant association was found (19).

The aim of this study was to examine the expression of PD-L1 on TC and TIC, and PD-1 on TIC, in chemoradiotherapy-naïve primary EG adenocarcinoma and paired lymph node metastases. Particular attention was given to their relationship with MMR status and prognosis. The prognostic value of PD-L1 and PD-1 expression at the mRNA level was also examined in 354 cases of gastric cancer and 161 cases of esophageal cancer in The Cancer Genome Atlas (TCGA). The association between CD8^+^ T cells and the expression of PD-L1 on TC and TIC, and PD-1 on TIC, in primary tumours was also investigated.

## Materials and Methods

### Study Design and Participants

The study cohort encompasses a previously described consecutive series of 174 patients with chemoradiotherapy-naïve esophageal and gastric adenocarcinoma (20–22). All patients were subjected to surgical resection at the University Hospitals of Lund and Malmö between January 1, 2006 and December 31, 2010. Tumour location was determined on endoscopy findings and classification of the tumour stage was done according to the 7th edition of the UICC/AJCC TNM classification, in which esophago-gastric junction tumours are classified as esophageal tumours (23). However, in clinical practice, and according to the new 8th edition of the UICC/AJCC TNM classification (24), esophago-gastric junction Siewert type 3 tumours are managed as gastric cancer, hence this definition is used in our subgroup analyses. Clinical data regarding recurrence and vital status were obtained retrospectively from medical records and the last update, with additional re-examination of some of the clinicopathological data, reaches until March 2016. Time to recurrence (TTR) was defined as time from diagnosis (date of result of the preoperative biopsy) to the date of proven recurrent disease (local, regional, or distant) by biopsy or radiology. TTR was not calculated for patients with distant metastases (M1) or macroscopic residual tumour (R2). OS was defined as time from diagnosis (date of result of the preoperative biopsy) to the date of death from any cause. Residual tumour status was classified as: R0 = no residual tumour (free resection margins according to pathology report), R1 = microscopic residual tumour (narrow or compromised resection margins according to pathology report), R2 = macroscopic residual tumour (according to the operative report). In the cohort, three patients had known M1-disease at the time of surgery, and underwent surgery with the aim to decrease symptoms from the primary tumour. The remainder of the cohort, 98.7%, was operated on with a curative intent, but in 16 of these patients, M1-disease was detected either during surgery or postoperatively through pathological examination of the resected specimen. Neoadjuvant or perioperative oncological therapy was not given to any of the patients in this cohort. Postoperative adjuvant treatment was given to 13 patients (7.5%), of which 11 received chemoradiotherapy, 1 received chemotherapy, and 1 received radiotherapy.

### Tissue Microarray Construction

Tissue microarrays (TMAs) were constructed as previously described using a semiautomated arraying device (TMArrayer, Pathology Devices, Westminister, MD, USA) (20). Duplicate tissue cores (1 mm) were obtained from two different blocks from all of the 174 primary tumours. Paired lymph node metastases were sampled from 81 cases, also in duplicate cores and from separate metastatic lymph nodes if more than one was present and of sufficient size.

### Immunohistochemical Staining and Evaluation

For immunohistochemical analysis of MMR proteins, 4 μm TMA sections were automatically pretreated and stained using the Benchmark Ultra Ventana platform. MMR status was evaluated using “ready to use,” RTU, monoclonal antibodies against MLH1 (Clone M1, Ventana/Roche, Basel, Schweiz,), PMS2 (Clone EPR3947, Ventana/Roche, Basel, Schweiz), MSH2 (Clone G219-1129, Ventana/Roche, Basel, Schweiz) and MSH6 using a monoclonal antibody (EPR3945, Nordic Biosite, Taby, Sweden) diluted 1:50. Immunohistochemical stainings were denoted as negative when all TCs showed loss of nuclear staining. Surrounding stromal cells and TICs served as internal controls for each TMA core. Tumour samples lacking nuclear staining of MLH1, PMS2, MSH2, or MSH6 were considered to have dMMR status. For immunohistochemical analyses of PD-1 and PD-L1, 4 μm tissue sections were pre-treated using the DAKO PT link system (DAKO; Glostrup, Copenhagen, Denmark) and stained in an Autostainer Plus (Dako; Glostrup, Denmark) with the anti PD-1 antibody NAT105, ab52587; Abcam, Cambridge, MA, USA, diluted 1:50 and the anti PD-L1 E1L3N, Cell Signaling Technology, Inc. (CST), Danvers, MA, USA) diluted 1:200. For scoring of PD-L1 and PD-1 expression the percentage of stained TIC was categorized as <10, 10–49, and 50–100%. For scoring of PD-L1 expression on TC the percentage of stained cells was categorized as <1, 1–4, 5–9, 10–49% and 50–100%, in accordance with a previous study by Berntsson et al. (25). The annotation of PD-L1 and PD-1 expression was carried out manually by two independent observers (MS, KJ), the latter being a board certified pathologist. Discrepant cases were re-evaluated and discussed in order to reach consensus. Only lymph nodes with presence of clearly visible metastatic tumour cells were evaluated for PD-L1 and PD-1 positive immune cells. The staining and automated analysis of CD8^+^ T cells has been described previously (21).

### EBER *in situ* Hybridization

For detection of Epstein-Barr virus (EBV)-infected tumours, the presence of EBV-encoded RNA (EBER) transcripts was analysed by chromogenic *in situ* hybridization on 4-μm-thick TMA sections with appropriate controls, performed on the Ventana Benchmark ULTRA automated platform (Ventana Medical Systems, Tucson, AZ, USA) following the manufacturer's protocol. The EBER 1 DNP probe (760-1209, Ventana Medical Systems) was used for detection, ISH iVIEW Blue Plus Detection kit (760-097, Ventana Medical Systems) was used to produce the chromogenic reaction, and slides were counterstained with Red Counterstain II (780-2218, Ventana Medical Systems).

### Survival Analysis Using TCGA Samples

Gastric and esophageal tumour samples were collected from TCGA project from the Genomic Data Commons (GDC). The fragments per kilobase of exon per million mapped reads (FPKM) values were retrieved and the average FPKM value was used for all individual samples for each tissue to estimate PD-L1 and PD-1 gene expression levels. A cut-off value of 1 FPKM was used as a detection limit across all tissues. The patients were classified into two groups and their prognoses examined based on the FPKM values. Genes with low expression were excluded i.e., those with a median expression among samples <1 during the analysis. The prognosis of each group of patients was examined by Kaplan-Meier survival estimates. To choose the best FPKM cut-offs for the most significant grouping of the patients, FPKM values from the 20 to 80th percentiles were used and significant differences in the survival outcomes of the groups were examined.

### Statistics

To evaluate associations between categories of PD-L1 expression on TC and TIC, and PD-1 expression on TIC, and clinicopathological factors, Kruskal–Wallis test was used for continuous variables and Chi-Square (linear-by-linear) test for categorical variables. Wilcoxon signed-rank test was applied to compare the distribution of PD-L1 and PD-1 expression in primary tumours and paired lymph node metastases. Kaplan-Meier analysis and the log rank test were applied to detect differences in TTR and OS. Cox proportional hazards models were used to calculate univariable and multivariable hazard ratios (HR) for TTR and OS. To assess the association between CD8^+^ T cells with PD-L1 expression on TIC in primary tumours, Mann-Whitney *U-*test was used.

The multivariable model included age, primary tumour location, adjuvant treatment, T stage, N stage, M stage, differentiation grade, residual tumour status, and MMR status. The proportional hazards assumption was tested using Cox regression with a time-dependent covariate analysis, whereby the proportional hazard assumption was considered to be satisfied when the factor x time interaction was non-significant.

All tests were two-tailed and *p* ≤ 0.05 were considered significant. All calculations were performed using IBM SPSS Statistics for Mac version 24.0 (IBM, Armonk, NY, USA).

## Results

### Distribution of PD-L1 and PD-1 Expression in Primary Tumours and Paired Lymph Node Metastases

**Figure 1:**
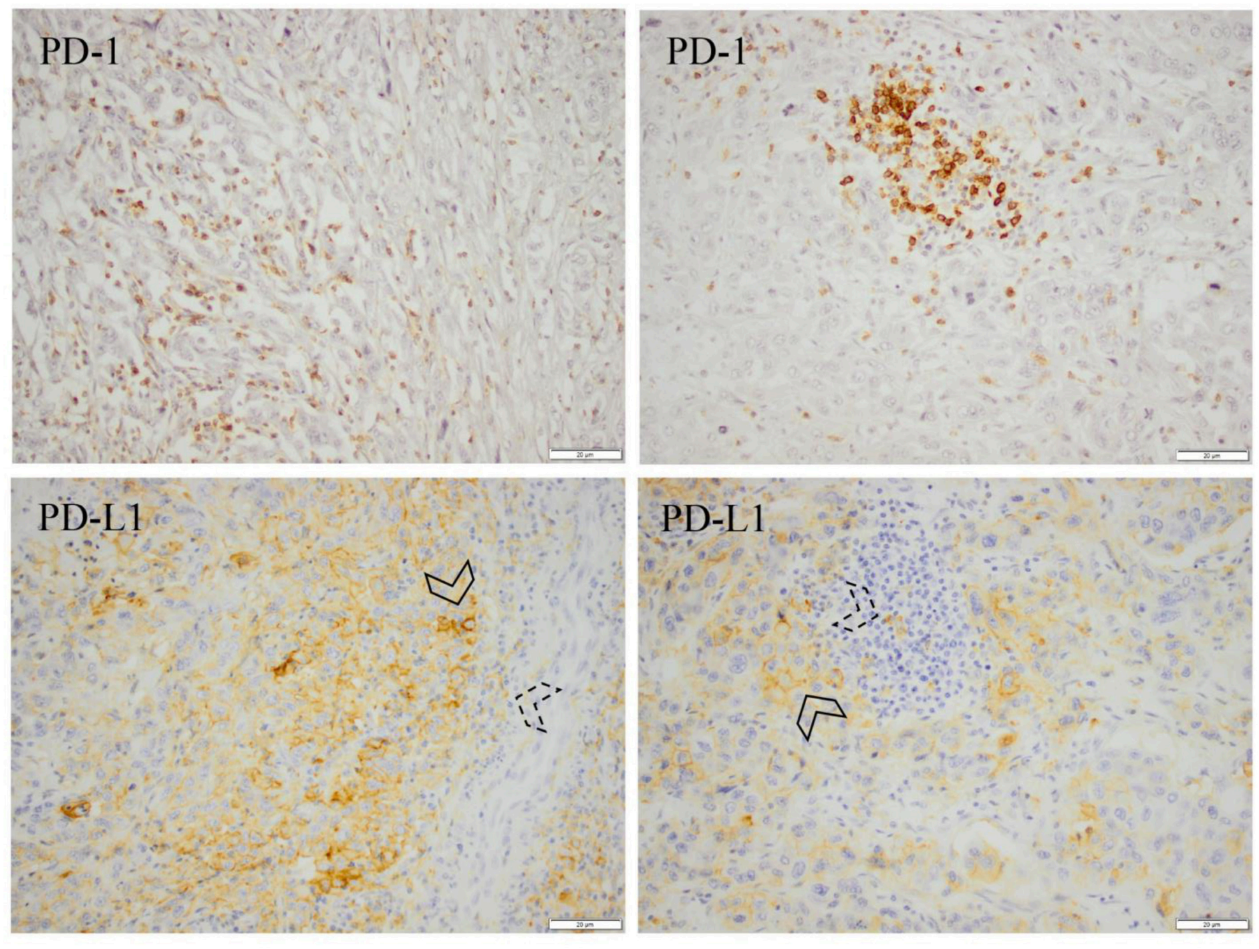
Immunohistochemical expression of PD-1 and PD-L1 in the primary tumour (left) and a paired lymph node metastasis (right) from an esophageal (cardia Siewert 1) pT3N3M0 tumour. PD-1 and PD-L1 positivity was denoted in >50% of the immune cells, and PD-L1 positivity was denoted in >50% of the tumour cells. Arrowheads with solid lines denote PD-L1 positive tumour cells and arrowheads with dashed lines denote PD-L1 positive immune cells. In general, PD-L1 positive immune cells in the lymph nodes were located in the vincinity of the metastatic deposits.

In primary tumours, PD-L1 expression on TC and TIC could be evaluated in 165/174 (94.8%) cases. Positive (≥ 1%) PD-L1 TC expression was denoted in 38 (23.0%) cases and positive (>10%) PD-L1 TIC expression in 68 (41.2%) cases. In paired lymph node metastases, PD-L1 expression on TC and TIC could be evaluated in 68/81 (84.0%) cases. Positive PD-L1 TC expression was denoted in 12 (17.6%) cases and positive PD-L1 TIC expression in 37 (54.4%) cases. In primary tumours, PD-1 expression on TIC could be evaluated in 170/174 (97.7%) cases. Positive (>10%) PD-1 TIC expression was denoted in 86 (50.6%) cases. In paired lymph node metastases, PD-1 expression on TIC could be evaluated in 75 (92.6%) cases. Positive PD-1 TIC expression was denoted in 40 (53.3%) cases. Sample immunohistochemical images are shown in Figure 1.

### Correlation Between PD-L1 and PD-1 Expression in Primary Tumours and Paired Lymph Node Metastases

**Figure 2:**
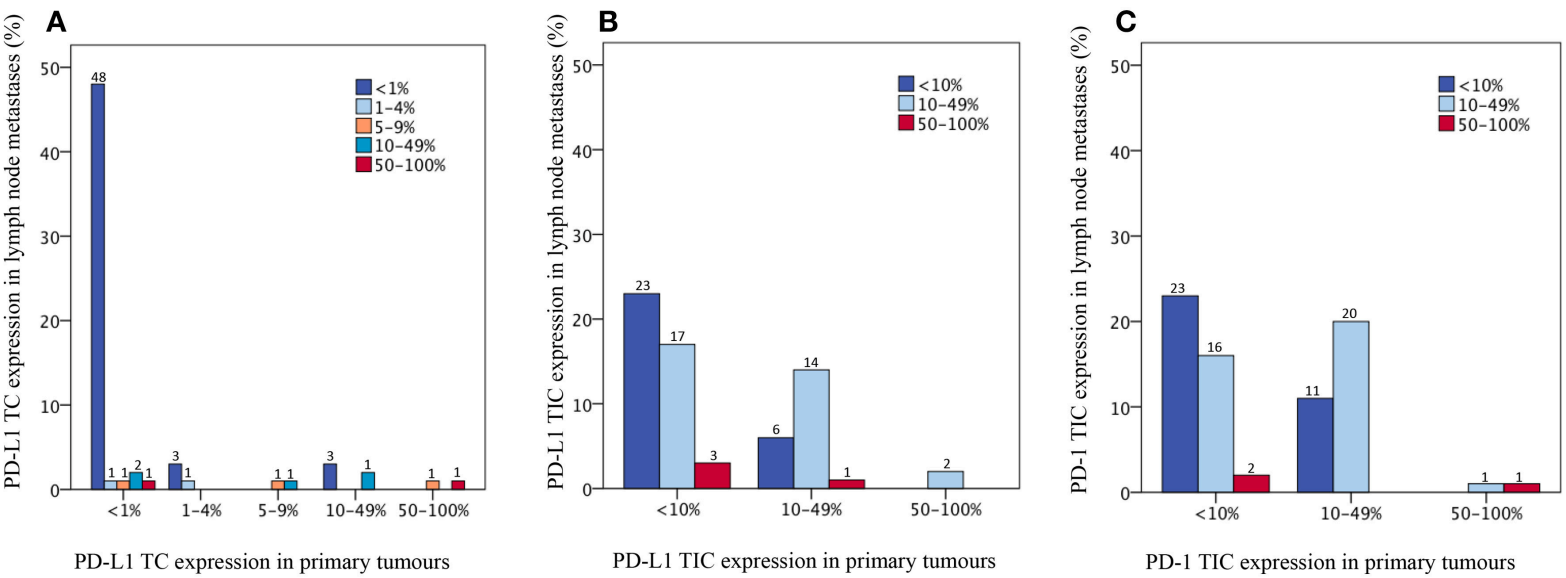
Bar charts visualising the distribution of PD-L1 expression on **(A)** tumour cells and **(B)** tumour-infiltrating immune cells and **(C)** the distribution of PD-1 expression on tumour-infiltrating immune cells, in primary tumours and paired lymph node metastases, in the entire cohort; **(A)** *p* = 1.000, **(B)** *p* = 0.009, **(C)** *p* = 0.180.

Bar charts visualising the distribution of PD-L1 and PD-1 expression in primary tumours and paired lymph node metastases, respectively, are shown in Figure 2. PD-L1 expression on TIC was significantly higher in lymph node metastases compared to primary tumours (*p* = 0.009). There was no significant difference in PD-L1 expression on TC or PD-1 expression on TIC between primary tumours and lymph node metastases.

### Associations With Clinicopathological Factors and Intercorrelation of PD-L1 and PD-1 Expression

The associations of PD-L1 and PD-1 expression with tumour and patient characteristics, as well as the intercorrelations of PD-L1 and PD-1 expression are shown in Table 1. Increased PD-L1 expression on TC was significantly associated with higher grade (*p* = 0.013) and dMMR (*p* < 0.001). Increased PD-L1 expression on TIC was significantly associated with lower T stage (*p* = 0.018), lower N stage (*p* = 0.001), lower M stage (*p* = 0.044) and dMMR (*p* < 0.001). Increased PD-1 expression on TIC was significantly associated with lower T stage (*p* = 0.049) and lower N stage (*p* = 0.027). Furthermore, there were moderate significant intercorrelations between PD-L1 and PD-1 expression on TC and TIC, and PD-1 expression on TIC, respectively (*p* < 0.001 for all, *R* = 0.312–0.412). The associations of PD-L1 and PD-1 expression with tumour and patient characteristics, as well as the intercorrelations of PD-L1 and PD-1 expression in esophageal and gastric cancer, respectively, are shown in Supplementary Tables S1, S2. Three (4.1%) out of 74 evaluable gastric cancers were positive for EBER mRNA, and all 95 evaluable esophageal cancers were negative. As shown in Supplementary Table S2, PD-L1 expression on TC was significantly higher in EBV-associated tumours, whereas PD-L1 or PD-1 expression on TIC did not differ by EBV status. Of note, all EBV-associated tumours had ≥ 10% PD-L1 positive TC (data not shown). As a common feature, all three EBV-associated tumours were located in the corpus, had pMMR status, were high-grade, radically resected and none had distant metastases. Two cases were of diffuse type and one of intestinal type according to the Laurén classification, two had N0 and one had N1 status, and the distribution of T stages was 2, 3, and 4. All three patients with EBV-associated tumours, one woman and two men, none of whom received adjuvant chemotherapy, were alive at last follow-up (data not shown).

**Table 1:** Associations with clinicopathological factors and intercorrelation of PD-L1 and PD-1 expression.

**Factor**	**PD-L1 Tumour cells**				**PD-L1 Immune cells**				**PD-1 Immune cells**			
n(%)	<1%	1–49%	≥50%	*P*	0–10%	11–50%	>50%	*P*	0–10%	11–50%	>50%	*P*
	127 (73.0)	33 (19.0)	5 (2.9)		97 (55.7)	56 (32.2)	12 (6.9)		84 (49.4)	80 (47.1)	6 (3.5)	
**AGE**												
Mean, median	69.2, 67.8	72.2, 72.9	76.4, 77.6	0.140	68.2, 67.7	71.7,71.5	76.4,80.6	0.028	70.5,71.4	70.0,69.3	75.6,77.8	0.425
(range)	(42.6–94.4)	(48.7–88.6)	(62.7–86.1)		(42.6–88.8)	(50.2–94.4)	(58.6–86.1)		(42.6–88.8)	(48.3–94.4)	(62.7,-83.1)	
**GENDER**												
Female	26 (20.5)	9 (27.3)	3 (60.0)	0.062	23 (23.7)	12 (21.4)	3 (25.0)	0.901	14 (16.7)	24 (30.0)	1 (16.7)	0.115
Male	101 (79.5)	24 (72.7)	2 (40.0)		74 (76.3)	44 (78.6)	9 (75.0)		70 (83.3)	56 (70.0)	5 (83.3)	
**T STAGE**												
T1	12 (9.4)	3 (9.1)	0 (0.0)	0.432	8 (8.2)	6 (10.7)	1 (8.3)	0.018	6 (7.1)	10 (12.5)	0 (0.0)	0.049
T2	21 (16.5)	8 (24.2)	1 (20.0)		13 (13.4)	12 (21.4)	5 (41.7)		10 (11.9)	20 (25.0)	2 (33.3)	
T3	71 (55.9)	19 (57.6)	4 (80.0)		56 (57.7)	32 (57.1)	6 (50.0)		53 (63.1)	38 (47.5)	4 (66.7)	
T4	23 (18.1)	3 (9.1)	0 (0.0)		20 (20.6)	6 (10.7)	0 (0.0)		15 (17.9)	12 (15.0)	0 (0.0)	
**N STAGE**												
N0	37 (29.1)	15 (45.5)	1 (20.0)	0.152	24 (24.7)	21 (37.5)	8 (66.7)	0.001	17 (20.2)	37 (46.3)	2 (33.3)	0.027
N1	20 (15.7)	7 (21.2)	2 (40.0)		14 (14.4)	14 (25.0)	1 (8.3)		19 (22.6)	9 (11.3)	1 (16.7)	
N2	36 (28.3)	3 (9.1)	1 (20.0)		28 (28.9)	10 (17.9)	2 (16.7)		22 (26.2)	19 (23.8)	0 (0.0)	
N3	34 (26.8)	8 (24.2)	1 (20.0)		31 (32.0)	11 (19.6)	1 (8.3)		26 (31.0)	15 (18.8)	3 (50.0)	
**M STAGE**												
M0	110 (86.6)	31 (93.9)	5 (100.0)	0.154	82 (84.5)	52 (92.9)	12 (100.0)	0.044	75 (89.3)	72 (90.0)	4 (66.7)	0.460
M1	17 (13.4)	2 (6.1)	0 (0.0)		15 (15.5)	4 (7.1)	0 (0.0)		9 (10.7)	8 (10.0)	2 (33.3)	
**GRADE**												
Low	49 (38.6)	7 (21.2)	0 (0.0)	0.013	29 (29.9)	22 (39.3)	5 (41.7)	0.206	26 (31.0)	32 (40.0)	0 (0.0)	0.861
High	78 (61.4)	26 (78.8)	5 (100.0)		68 (70.1)	34 (60.7)	7 (58.3)		58 (69.0)	48 (60.0)	6 (100.0)	
**RESIDUAL TUMOUR STATUS**												
R0	84 (66.1)	22 (66.7)	4 (80.0)	0.660	60 (61.9)	39 (69.6)	11 (91.7)	0.058	55 (65.5)	56 (70.0)	4 (66.7)	0.883
R1	36 (28.3)	9 (27.3)	1 (20.0)		31 (32.0)	14 (25.0)	1 (8.3)		26 (31.0)	18 (22.5)	2 (33.3)	
R2	7 (5.5)	2 (6.1)	(0.0)		6 (6.2)	3 (5.4)	0 (0.0)		3 (3.6)	6 (7.5)	0 (0.0)	
**LOCATION**												
Esophagus	75 (59.1)	18 (54.5)	2 (40.0)	0.390	50 (51.5)	37 (66.1)	8 (66.7)	0.083	52 (61.9)	41 (51.3)	3 (50.0)	0.176
Stomach	52 (40.9)	15 (45.5)	3 (60.0)		47 (48.5)	19 (33.9)	4 (33.3)		32 (38.1)	39 (48.8)	3 (50.0)	
**LAURÉN**												
Intestinal	87 (68.5)	22 (66.7)	4 (80.0)	0.894	63 (64.9)	40 (71.4)	10 (83.3)	0.255	58 (69.0)	54 (67.5)	4 (66.7)	0.709
Mixed	7 (5.5)	2 (6.1)	0 (0.0)		7 (7.2)	2 (3.6)	0 (0.0)		5 (6.0)	4 (5.0)	0 (0.0)	
Diffuse	33 (26.0)	9 (27.3)	1 (20.0)		27 (27.8)	14 (25.0)	2 (16.7)		21 (25.0)	22 (27.5)	2 (33.3)	
**MMR STATUS**												
pMMR	124 (97.6)	25 (75.8)	3 (60.0)	<0.001	95 (97.9)	49 (87.5)	8 (66.7)	<0.001	78 (92.9)	74 (92.5)	4 (66.7)	0.233
dMMR	3 (2.4)	8 (24.2)	2 (40.0)		2 (2.1)	7 (12.5)	4 (33.3)		6 (7.1)	6 (7.5)	2 (33.3)	
**PD-L1 TUMOUR CELLS**												
<1%	–	–	–		89 (91.8)	31 (55.4)	7 (58.3)	<0.001	71 (88.8)	53 (68.8)	1 (16.7)	<0.001
1–49%	–	–	–		7 (7.2)	24 (42.9)	2 (16.7)		7 (8.8)	22 (28.6)	4 (66.7)	
≥50%	–	–	–		1 (1.0)	1 (1.8)	3 (25.0)		2 (2.5)	2 (2.6)	1 (16.7)	
**PD-L1 IMMUNE CELLS**												
0–10%	89 (70.1)	7 (21.2)	1 (20.0)	<0.001	–	–	–		61 (76.3)	33 (42.9)	1 (16.7)	<0.001
11–50%	31 (24.4)	24 (72.7)	1 (20.0)		–	–	–		17 (21.3)	35 (45.5)	4 (66.7)	
>50%	7 (5.5)	2 (6.1)	3 (60.0)		–	–	–		2 (2.5)	9 (11.7)	1 (16.7)	
**PD-1 IMMUNE CELLS**					61 (64.2)	17 (30.4)	2 (16.7)	<0.001				
0–10%	71 (56.8)	7 (21.2)	2 (40.0)	<0.001	33 (34.7)	35 (62.5)	9 (75.0)		–	–	–	
11–50%	53 (42.4)	22 (66.7)	2 (40.0)		1 (1.1)	4 (7.1)	1 (8.3)		–	–	–	
>50%	1 (0.8)	4 (12.1)	1 (20.0)						–	–	–	

*MMR, mismatch repair; pMMR, mismatch repair proficiency; dMMR, mismatch repair deficiency*.

### Prognostic Significance of PD-L1 Expression on TC and TIC and PD-1 Expression on TIC in Primary Tumours

**Figure 3:**
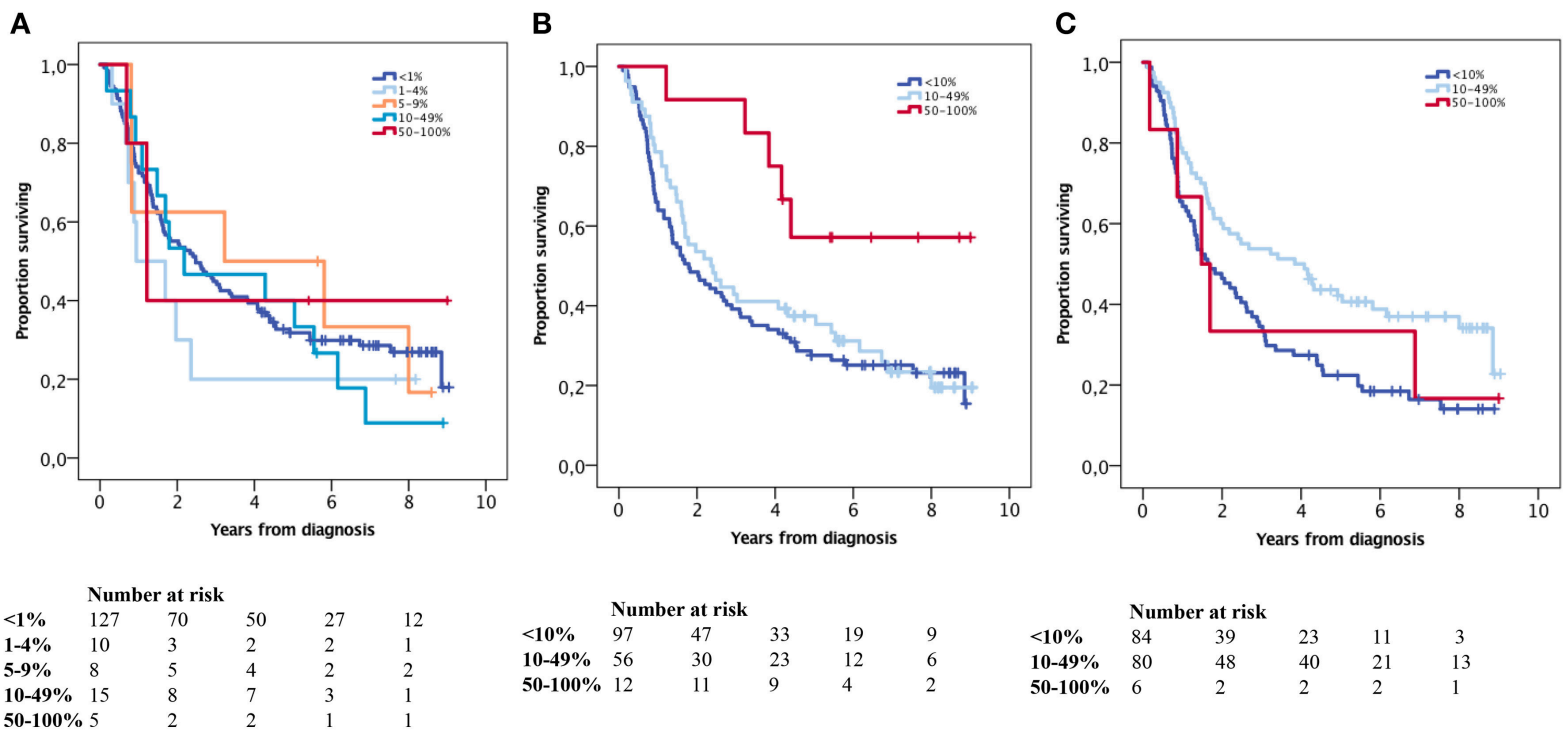
Kaplan-Meier analysis of OS in strata according to immunohistochemical staining categories of **(A)** PD-L1 expression on, **(B)** PD-L1 expression on, and **(C)** PD-1 expression on in the entire cohort. **(A)**<1% = ref, 1–4% *p* = 0.285, 5–9% *p* = 0.815, 10–49% *p* = 0.721, 50–100% *p* = 0.763, **(B)**<10% = ref, 10–49% *p* = 0.512, 50–100% *p* = 0.014, **(C)**<10% = ref, 10–49% *p* = 0.003, 50–100% *p* = 0.842.

**Figure 4:**
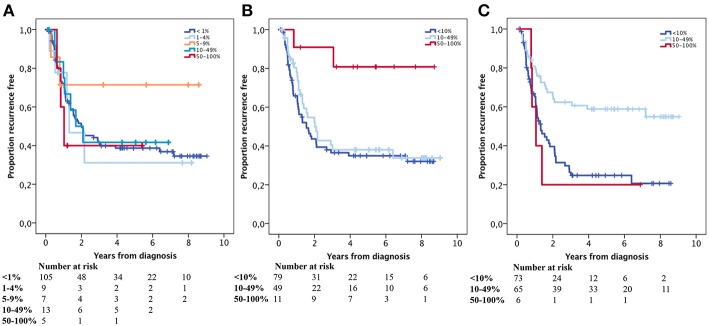
Kaplan-Meier estimates of TTR in strata according to immunohistochemical staining categories of **(A)** PD-L1 expression on, **(B)** PD-L1 expression on and **(C)** PD-1 expression on in the entire cohort. **(A)** <1% = ref, 1–4% *p* = 0.803, 5–9% *p* = 0.244, 10–49% *p* = 0.815, 50–100% *p* = 0.603, **(B)** <10% = ref, 10–49% *p* = 0.484, 50–100% *p* = 0.010, **(C)** <10% = ref, 10–49% *p* = 0.000, 50–100% *p* = 0.824.

Kaplan-Meier analyses of the prognostic impact of PD-L1 expression on TC and TIC and PD-1 expression on TIC are shown in Figure 3. High PD-L1 and PD-1 expression on TIC was significantly associated with a prolonged OS (*p* = 0.023 and *p* = 0.004, respectively). PD-L1 expression on TC was not prognostic. Similar results were found for TTR and are shown in Figure 4. For Cox regression analyses, PD-L1 expression on TIC was dichotomised at 0–49 vs. ≥ 50% and PD-1 expression was dichotomised at 0–9 vs. ≥ 10%. The time-dependent covariate was non-significant for both PD-L1 and PD-1 expression, and therefore, the factor x time interaction term was dropped from the model. The proportional hazard assumption was also considered to be satisfied with graphical evaluation using log-minus-log plots (data not shown). As shown in Table 2, the prognostic value regarding OS was confirmed for both PD-L1 and PD-1 on TIC in univariable analysis (HR = 0.35, 95% confidence interval [CI] = 0.14–0.87, and HR = 0.60, 95% CI = 0.42–0.85, respectively), and remained significant for PD-L1 but not PD-1 in multivariable analysis (HR = 0.39, 95% CI = 0.15–0.99). As shown in Table 3, both PD-L1 and PD-1 expression on TIC remained prognostic also regarding TTR in univariable analysis (HR = 0.20, CI = 0.05–0.80, and HR = 0.44, CI = 0.28–0.70, respectively), but not in multivariable analyses.

**Table 2:** Cox proportional hazards analysis of the impact of PD-L1 and PD-1 immune cell density on overall survival in the entire cohort.

	**OS**		
	***n* (events)**	**HR (95 % CI)**	***P***
**PD-L1**			
**Univariable**			
Low	153 (116)	1.00	
High	12 (5)	0.35 (0.14–0.87)	0.023
Multivariable			
Low	153 (116)	1.00	
High	12 (5)	0.39 (0.15–0.99)	0.048
**PD-1**			
**Univariable**			
Low	84 (70)	1.00	
High	86(56)	0.60 (0.42–0.85)	0.004
**Multivariable**			
Low	84 (70)	1.00	
High	86 (56)	0.70 (0.46–1.05)	0.086

*OS, overall survival; HR, hazard ratio; CI, confidence interval. Multivariable analysis includes age, location, adjuvant treatment, T stage, N stage, M stage, differentiation grade (high vs. low), resection margins (R0,R1,R2), mismatch repair status*.

**Table 3:** Cox proportional hazards analysis of the impact of PD-L1 and PD-1 immune cell density on time to recurrence in the entire cohort.

	**TTR**		
	***n* (events)**	**HR (95 % CI)**	***P***
**PD-L1**			
**Univariable**			
Low	128 (77)	1.00	
High	11 (2)	0.20 (0.05–0.80)	0.023
**Multivariable**			
Low	128 (77)	1.00	
High	11 (2)	0.75 (0.22–2.58)	0.647
**PD-1**			
**Univariable**			
Low	73 (50)	1.00	
High	71 (30)	0.44 (0.28–0.70)	0.001
**Multivariable**			
Low	73 (50)	1.00	
High	71 (30)	0.87 (0.52–1.46)	0.602

*TTR, time to recurrence; HR, hazard ratio; CI, confidence interval. Multivariable analysis includes age, location, adjuvant treatment, T stage, N stage, M stage, differentiation grade (high vs. low), resection margins (R0,R1,R2), mismatch repair status*.

### Prognostic Impact of PD-L1 and PD-1 mRNA Expression

**Figure 5:**
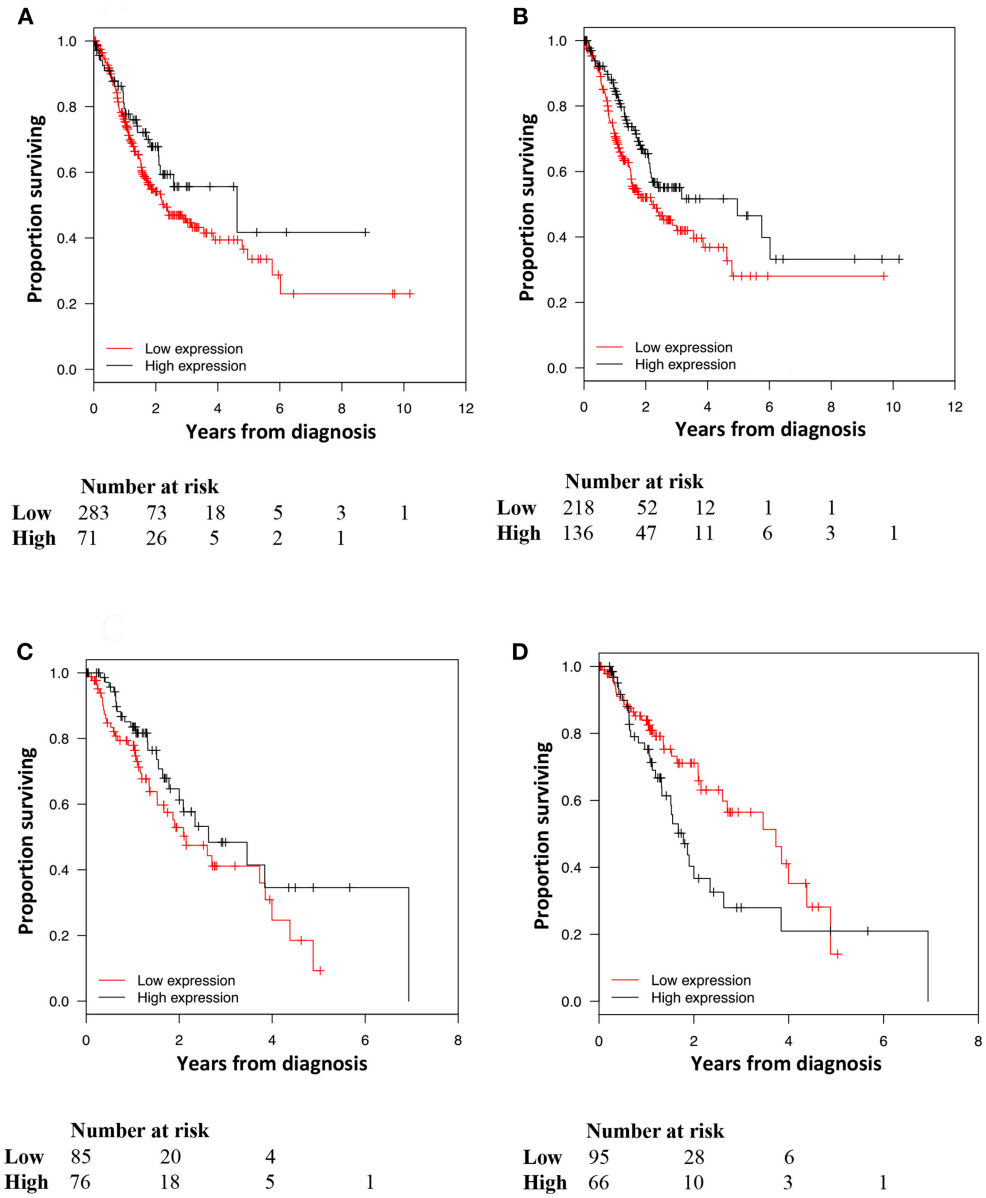
Kaplan-Meier estimates of OS in strata according to high and low **(A)** PD-L1 and **(B)** PD-1 mRNA expression in gastric cancer and high and low **(C)** PD-L1 and **(D)** PD-1 mRNA expression, in esophageal cancer, in TCGA. **(A)** *p* = 0.116, **(B)** *p* = 0.041, **(C)** *p* = 0.168, **(D)** *p* = 0.053.

Kaplan-Meier analyses of OS according to PD-L1 and PD-1 mRNA expression in 354 cases of gastric cancer and 161 cases of esophageal cancer in TCGA are shown in Figure 5. PD-L1 expression was not prognostic at the transcript level in neither esophageal nor gastric cancer. High PD-1 expression was significantly associated with a prolonged OS in gastric cancer (*p* = 0.041), whereas a borderline significant association was observed between high PD-1 expression and a shorter OS (*p* = 0.053) in esophageal cancer. Of note, both squamous cell carcinoma (SCC) and adenocarcinoma are included in the esophageal cancer dataset in TCGA.

### Associations of MMR Status With Clinicopathological Factors and Prognosis

In the entire cohort, 14 (8.1%) primary tumours had dMMR status. The distribution of these cases according to anatomical subsite was; 3 (7.5%) in the lower third of the esophagus including esophago-gastric junction (Siewert type 1), 3 (13.0%) in the true cardia (Siewert type 2), 1 (11.1%) in the subcardial stomach (Siewert type 3), 1 (50.0%) in fundus, 3 (8.3%) in corpus, 2 (8.7%) in antrum and 1 (20.0%) in pylorus.

**Figure 6:**
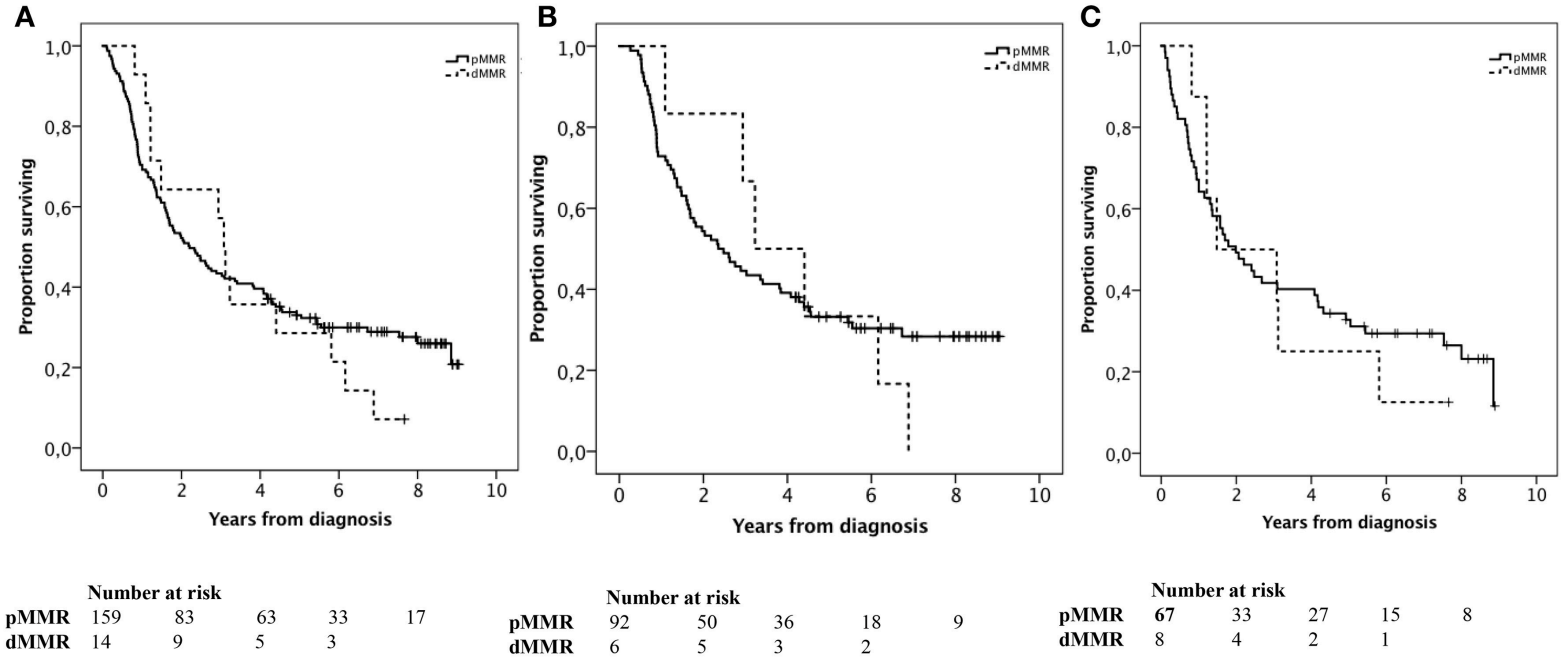
Kaplan-Meier estimates of OS in strata according to MMR status in **(A)** the entire cohort, *p* = 0.626, **(B)** esophageal tumours, *p* = 0.824, and **(C)** gastric tumours, *p* = 0.740.

Associations of MMR status with tumour and patient characteristics are shown in Supplementary Table S3. dMMR was significantly associated with higher age (*p* = 0.001) and lower N stage (*p* = 0.012). Kaplan-Meier analyses of the prognostic impact of MMR status are shown in Figure 6. Similar and non-significant results were found for TTR (data not shown).

### Associations of CD8^+^ T Cells With PD-L1 Expression on TC and TIC and PD-1 Expression on TIC in Primary Tumours

**Figure 7:**
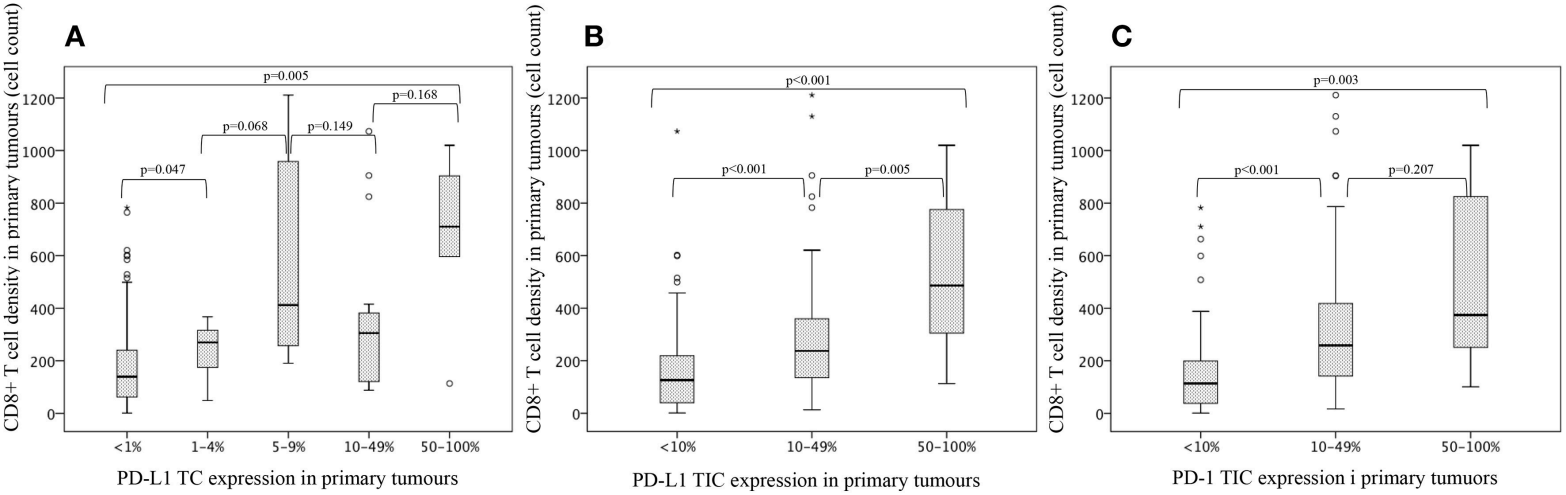
Box plots visualising the associations of CD8^+^ T cells with **(A)** PD-L1 expression on tumour cells and **(B)** PD-L1 expression on tumour-infiltrating immune cells and **(C)** PD-1 expression on tumour-infiltrating immune cells, in primary tumours in the entire cohort.

The associations of CD8^+^ T cells with PD-L1 expression on TIC in primary tumours in the entire cohort are shown in Figure 7. There was a significant stepwise positive association between CD8^+^ T cell density and categories of in particular PD-L1, but also PD-1, expression, on TIC. The associations between CD8^+^ T cells and PD-L1 on TC were less evident.

## Discussion

This is one of few studies that has investigated the intercorrelation between PD-L1 and PD-1 expression in primary tumours and paired lymph node metastases, as well as the relationship between MMR status and prognosis, in esophageal adenocarcinoma.

High expression of both PD-L1 and PD-1 on TIC was significantly associated with a prolonged OS, the former independently of other prognostic factors and MMR status. These findings are partly in line with the study by Böger et al. on gastric cancer wherein patients with high expression of PD-L1 on TIC had a significantly better OS in both univariable and multivariable analysis and patients with high expression of PD-L1 on TC had a significantly improved survival in univariable analyses. However, patients with high PD-L1 expression on both TC and TIC had the best OS (13). Of note, the present study and the study by Böger et al. utilized the same monoclonal antibody for PD-L1 staining. In contrast, other studies have demonstrated associations between positive PD-L1 expression and poor survival, e.g., Zhang et al. (15). Of note, all tumours in the present study as well as in the study by Böger et al. (13) and Zhang et al. (15) were derived from patients who did not receive neoadjuvant therapy, which makes the cohorts comparable.

Regarding PD-1 expression and its association with prognosis, only a few studies have investigated this in EG cancer, and no significant associations have been found besides from one Asian study on gastric cancer by Gao et al. (*n* = 119) wherein PD-1 expression on CD8^+^ T cells was found to be an unfavourable prognostic factor, but no other types of immune cells were explored regarding PD-1 expression (26). In the study by Böger et al. patients with PD-1 positive gastric tumours had a better tumour specific survival, however only borderline significant (13). In another study on chemoradiotherapy-naïve esophageal cancer (*n* = 354), PD-1 positivity correlated with increased mortality, but not after adjusting for other prognostic factors (27).

The expression of PD-L1 and PD-1 on TC and TIC in this study is well in line with the above mentioned study by Böger et al. (13). Furthermore, PD-L1 expression on TIC was found to be significantly higher in lymph node metastases compared to primary tumours, whereas the expression of PD-L1 on TC and PD-1 on TIC did not differ between primary tumours and lymph node metastases. The previously mentioned study on gastric cancer, by Böger et al. showed a concordance between the expression of PD-L1 on TC and TIC and PD-1 on TIC in liver metastases (*n* = 15) and primary tumours (13). In the study on gastric cancer by Gao et al. the positive rate of PD-L1 expression on TC was found to be higher in metastatic lymph nodes (*n* = 119) than in primary tumours, TIC not investigated (26). Of note, the present study and the study by Gao et al. utilized the same monoclonal antibody for PD-L1 staining. Furthermore, Dislich et al. analysed PD-L1 expression on TC and TIC in primary esophageal adenocarcinoma (*n* = 112), paired lymph node metastases (*n* = 55) and distant metastases (*n* = 17), and concluded that the expression in the different entities “does not necessarily correlate” (19). The expression of PD-L1 in primary tumours and lymph node metastases has also been investigated in some other types of cancer, with divergent results. In a study by Li et al. (*n* = 101) PD-L1 expression was compared between primary triple negative breast tumours and axillary lymph nodes (*n* = 101), whereby PD-L1 expression was found to be significantly higher in both lymphocytes and TC of the axillary lymph node metastases compared to the primary tumours (28). On the other hand, in a study by Heeren et al. comparing PD-L1 expression in primary tumours (*n* = 205) and paired lymph node metastases (*n* = 127) in cervix squamous cell carcinoma and adenocarcinoma no significant difference was found (29).

The findings from our study, as well as from the study by Böger et al. (13) and Gao et al. (26) may be of potential clinical relevance as they suggest that analysis of a biopsy from a metastasis may also provide prognostic or predictive information. Furthermore, the results in our study reflects the heterogeneity of PD-L1 expression between primary tumours and lymph node metastases, which is in line with other previous studies (26, 30, 31). The findings may also contribute predictive information in cases where treatment with immune-checkpoint blockade targeting the PD-1/PD-L1 pathway is considered, provided PD-L1 expression can be implemented as a predictive biomarker for such therapy. However, before this can be implemented in clinical protocols, further studies are needed to investigate how neoadjuvant chemotherapy or chemoradiotherapy may affect the quantity of PD-L1 and PD-1 positive cells in the tumour microenvironment (TME).

To validate our data, the prognostic value of PD-L1 and PD-1 expression was also examined at the mRNA level in 354 cases of gastric cancer and 161 cases of esophageal cancer in TCGA. PD-L1 mRNA levels were not found to confer any prognostic information. This is however not surprising, as our data clearly demonstrate a prognostic value only for PD-L1 expression on TIC and not on TC, and mRNA levels of PD-L1 represent a mixture of TIC and TC. Regarding PD-1, high mRNA expression was found to be significantly associated with a prolonged OS in gastric cancer, thus validating the immunohistochemical data, whereas a borderline significant trend towards an association with a shorter OS was seen in esophageal cancer. A possible explanation for the contrasting findings in gastric and esophageal cancer could be that TCGA esophageal cancer dataset includes both SCC and adenocarcinoma, which may cloud the results, as esophageal SCC and adenocarcinoma have distinct and divergent molecular characteristics (32).

In this study, no significant associations were found between MMR status and clinical outcome. This is in line with some previous studies, for example the study on esophageal adenocarcinoma by Dislich et al. (19) and a meta-analysis on gastric cancer by Polom et al. (33), but as mentioned before, the number of studies on this topic is sparse and the results are contrasting (16, 17, 33, 34).

The present study demonstrates a significant association between dMMR tumours and an increased PD-L1 expression on TC and/or TIC which is consistent with a study on gastric cancer by Kawazoe et al. (*n* = 487), wherein PD-L1 positivity (≥ 1%) on both TC and TIC was more frequently observed in dMMR tumours (35). The patients included in the Kawazoe study did not receive any chemotherapy before surgery which makes the cohorts comparable. In the study by Böger et al. PD-L1 expression on TC but not on TIC was found to correlate with dMMR (13).

Furthermore, it could be hypothesized that the beneficial prognostic outcome for patients with tumours displaying high expression of PD-L1 on TIC, is due to the significant positive association of CD8^+^ T cell density and PD-L1 expression on TIC. Regarding gastric cancer, an Asian study by Wang et al. (*n* = 509) demonstrated that patients with positive (>5%) PD-L1 TC expression and high CD8^+^ T cell infiltration was associated with an improved OS, and positive PD-L1 status correlated with high CD8^+^ T cell infiltration. PD-L1 expression on TIC was not investigated (36). Similar results were found in the study by Kawazae et al. wherein positive PD-L1 expression on both TC and TIC was investigated and found to be significantly associated with high CD8^+^ T cell density. High density of CD8^+^ T cells was also found to be an independent beneficial prognostic factor for better survival (35). On the other hand, in the above mentioned study by Gao et al. PD-L1 expression on TC and a high density of CD8^+^ T cells were prognostic factors for shorter disease-free survival, furthermore PD-L1 expression on TC was found to be associated with CD8^+^ T cell density in the primary tumour (26). It must however be kept in mind that the TME is highly complex, with several undiscovered interactions. Furthermore, IHC only mirrors a temporary reflection of its dynamic landscape. The classification of tumours into different TME subgroups has been suggested on the basis of PD-L1 expression, and the presence or absence, of tumour-infiltrating lymphocytes (TILs), including CD8^+^ T cells (37). This classification was initially proposed in melanoma by Teng et al. (37) but has recently also been applied in gastric tumours in a study by Cho et al. (*n* = 247) wherein the PD-L1 TC positive/TIL positive TME subgroup, was found to be more frequent in EBV-positive and MSI-H gastric tumours and also to be associated with a favourable prognosis (38). Of note, 50% of EBV-positive and 60% of MSI-H tumours demonstrated high levels of PD-L1 expression (38). Several studies, including the study by Teng et al. and Cho et al. have discussed the putative mechanism by which the TCs enhance their PD-L1 expression due to external stimuli from TILs e.g., CD8 ^+^ T cells. (38–42). Taken together it has been proposed that interferon-gamma (IFNγ), which is secreted by activated CD8^+^ T cells, upregulates the PD-L1 TC expression and hence, TCs escape immune surveillance by paralyzing and disabling the CD8^+^ T cells to attack (40, 41). However, CD8^+^ T cells themselves are also promoted by IFNγ (42) which could mean that if we reach a positive balance in the TME wherein the beneficial effects of IFNγ bridges the unfavourable effects of PD-L1, the net contribution may be an improved tumour surveillance. Accordingly, this could potentially strengthen the hypothesis that the beneficial prognostic value of high PD-L1 TIC expression in EG adenocarcinoma is due to its positive association with CD8^+^ T cell density, as demonstrated in the present study. In the study by Mimura et al. it is proposed that gastric tumours with CD8^+^ T cells in the TME would be more susceptible to PD-1/PD-L1 blockade (40). Possibly, the higher expression of PD-L1 in lymph nodes as compared to primary tumors may also have a relation to the presence of IFNγ producing CD8^+^ T cells and other immune cells being accumulated in lymph nodes.

A possible limitation to this study is the known heterogeneity of PD-L1 expression. For example, in a study on non-small cell lung cancer (*n* = 160), lung biopsies and corresponding tumours were compared regarding PD-L1 expression, and the expression was found to be lower in the biopsy compared to the resected tumour in all cases, in particular the expression on TIC (43). For gastric cancer, the heterogeneity of PD-L1 was investigated in a study by Wang et al. (*n* = 550) also demonstrating lower expression in TMA cores compared to the whole tissue blocks, although the use of two cores, one central and one from the invasive front, was thought to minimize the heterogeneity issue (44). In our study, duplicate tissue cores were also obtained from two different blocks from all of the 174 primary tumours, and from the paired lymph node metastases duplicate cores were sampled from separate metastatic lymph nodes if more than one was present and of sufficient size. Moreover, in a recent study on colorectal cancer, a good concordance was demonstrated between PD-L1 and PD-1 expression in TMA cores compared to whole tissue sections (25). Another limitation that could be argued is the use of different PD-L1 antibodies in different studies. We utilized the platform independent clone E1L3N, which according to a recent study on primary and metastatic bladder cancer (*n* = 156) is comparable to the 3 FDA-approved clones 22C3, 28.8 and SP142 (45).

The frequency of EBV-associated gastric tumours in the herein analysed cohort (4.1%) is well in line with a study by Böger et al. (*n* = 484), wherein 5.0% of all gastric adenocarcinomas were found to be EBV-associated (46). According to a meta-analysis by Murphy et al. around 8% of all gastric tumours are EBV-positive (47). These tumours mostly occur in the proximal part of the stomach (47–49) and are associated with an elevated PD-L1 TC and /or TIC expression (13, 48). In the present study, only PD-L1 expression on TC was significantly higher in the small number of EBV-associated tumours. Moreover, as also shown in this study, patients with EBV-associated tumours have an improved survival (50) and, in addition, they appear to benefit from PD-1/PD-L1 blockade (46, 48).

In conclusion, the results from this study demonstrate that PD-L1 expression on TIC is higher in lymph node metastases compared to primary tumours, correlates with dMMR, and is an independent factor of prolonged survival in patients with chemoradiotherapy-naïve EG adenocarcinoma. The prognostic value of PD-1 expression was only evident in unadjusted analysis and PD-L1 expression on TC did not confer any prognostic value. Given the cell-type specific prognostic impact of PD-L1 expression, assessment of PD-L1 mRNA expression is not likely to be a useful tool in the clinical setting. Taken together, these findings suggest that PD-L1 expression on TIC may be a useful biomarker for identifying patients who may not benefit from additional chemo-or chemoradiotherapy before curative surgery, and in the future, the prognostic information can also be of great interest when considering treatment with immune-checkpoint blockade targeting the PD-1/PD-L1 pathway, provided that PD-L1 expression can be implemented as a predictive biomarker for such therapy.

## Data Availability

All data generated or analysed during this study are included in this published article.

## Ethics Statement

All EU and national regulations and requirements for handling human samples have been fully complied with during the conduct of this project; i.e., decision no. 1110/94/EC of the European Parliament and of the Council (OJL126 18,5,94), the Helsinki Declaration on ethical principles for medical research involving human subjects, and the EU Council Convention on human rights and Biomedicine. Approval for the study was obtained from the Ethics committee of Lund University (ref no 445/07), whereby the committee waived no need for consent other than the option to opt out.

## Author Contributions

KJ conceived and designed the experiments. MS, CZ, and AM performed the experiments. MS, KJ, CZ, and AM analysed the data. DB, CH, AM, MU, BN, and KL contributed reagents, materials, and analysis tools. MS and CZ prepared the figures and tables. MS and KJ wrote the paper.

### Conflict of Interest Statement

The authors declare that the research was conducted in the absence of any commercial or financial relationships that could be construed as a potential conflict of interest.

## Abbreviations

EG: esophageal and gastric
OS: overall survival
mAb: monoclonal antibodies
HER2: human epidermal growth factor receptor 2
VEGFR2: vascular endothelial growth factor receptor 2
PD-1: programmed death receptor 1
PD-L1: programmed death ligand 1
TC: tumour cells
TIC: tumour-infiltrating immune cells
dMMR: mismatch repair deficiency
MSI: microsatellite instability
MSI-H: MSI-High
ORR: objective response rate
MMR: mismatch repair
MAGIC: Medical Research Council Adjuvant Gastric Infusional Chemotherapy
TCGA: The Cancer Genome Atlas
TTR: time to recurrence
TMAs: tissue microarrays
EBV: Epstein-Barr virus
EBER: EBV-encoded RNA
GDC: Genomic Data Commons
FPKM: The fragments per kilobase of exon per million mapped reads
HR: hazard ratio
CI: confidence interval
SCC: squamous cell carcinoma
TME: tumour microenvironment
TILs: tumour-infiltrating lymphocytes
IFNγ: interferon-gamma.
