# Supplementary material for: Expression of PD-L1 and PD-1 in Chemoradiotherapy-Naïve Esophageal and Gastric Adenocarcinoma: Relationship With Mismatch Repair Status and Survival
Source: Front Oncol. 2019 Mar 13;9:136. doi: 10.3389/fonc.2019.00136 (PMC6425870; doi:10.3389/fonc.2019.00136)
Supplement: Supplementary file 1 [file Table_1.docx]

**Supplementary Table S1. Associations with clinicopathological factors and intercorrelation of PD-L1 and PD-1 expression in esophageal cancer**

| **Factor** | **PD-L1 Tumour cells** | | | | **PD-L1 Immune cells** | | | | **PD-1 Immune cells** | | | |
| --- | --- | --- | --- | --- | --- | --- | --- | --- | --- | --- | --- | --- |
| n(%) | <1% | 1-49% | ≥50% | *P* | 0-10% | 11-50% | >50% | *P* | 0-10% | 11-50% | >50% | *P* |
| **Age** |  |  |  |  |  |  |  |  |  |  |  |  |
| mean, median  (range) | 68.3, 65.9  (48.2-88.6) | 68.3, 65.9  (48.2-88.6) | 68.3, 68.3  (62.7-74.0) | 0.770 | 66.5, 65.1  (48.2-84.9) | 69.3, 68.7  (50.2-88.6) | 72.9, 80.0  (58.6-85.3) | 0.256 | 68.2, 67.0  (48.2-88.5) | 68.5, 65.9  (48.4-88.6) | 71.6, 69,2  (62.7-83.1) | 0.842 |
| **Gender** |  |  |  |  |  |  |  |  |  |  |  |  |
| Female | 8(10.7) | 4(22.2) | 1(50.0) | 0.058 | 7(14.0) | 6(16.2) | 0(0.0) | 0.566 | 4(7.7) | 9(22.0) | 0(0.0) | 0.163 |
| Male | 67(89.3) | 14(77.8) | 1(50.0) |  | 43(86.0) | 31(83.8) | 8(100.0) |  | 48(92.3) | 32(78.0) | 3(100.0) |  |
| **T stage** |  |  |  |  |  |  |  |  |  |  |  |  |
| T1 | 6(8.0) | 1(5.6) | 0(0.0) | 0.857 | 3(6.0) | 3(8.1) | 1(12.5) | 0.038 | 4(7.7) | 4(9.8) | 0(0.0) | 0.161 |
| T2 | 11(14.7) | 4(22.2) | 0(0.0) |  | 5(10.0) | 6(16.2) | 4(50.0) |  | 3(5.8) | 12(29.3) | 0(0.0) |  |
| T3 | 49(65.3) | 12(66.7) | 2(100.0) |  | 36(72.0) | 24(64.9) | 3(37.5) |  | 39(75.0) | 21(51.2) | 3(100.0) |  |
| T4 | 9(12.0) | 1(5.6) | 0(0.0) |  | 6(12.0) | 4(10.8) | 0(0.0) |  | 6(11.5) | 4(9.8) | 0(0.0) |  |
| **N stage** |  |  |  |  |  |  |  |  |  |  |  |  |
| N0 | 14(18.7) | 7(38.9) | 0(0.0) | 0.292 | 6(12.0) | 10(27.0) | 5(62.5) | 0.001 | 5(9.6) | 15(36.6) | 1(33.3) | 0.039 |
| N1 | 11(14.7) | 4(22.2) | 1(50.0) |  | 6(12.0) | 10(27.0) | 0(0.0) |  | 11(21.2) | 6(14.6) | 0(0.0) |  |
| N2 | 27(36.0) | 1(5.6) | 0(0.0) |  | 19(38.0) | 7(18.9) | 2(25.0) |  | 17(32.7) | 11(26.8) | 0(0.0) |  |
| N3 | 23(30.7) | 6(33.3) | 1(50.0) |  | 19(38.0) | 10(27.0 | 1(12.5) |  | 19(36.5) | 9(22.0) | 2(66.7) |  |
| **M stage** |  |  |  |  |  |  |  |  |  |  |  |  |
| M0 | 68(90.7) | 17(94.4) | 2(100.0) | 0.507 | 45(90.0) | 34(91.9) | 8(100.0) | 0.407 | 49(94.2) | 37(90.2) | 2(66.7) | 0.172 |
| M1 | 7(9.3) | 1(5.6) | 0(0.0) |  | 5(10.0) | 3(8.1) | 0(0.0) |  | 3(5.8) | 4(9.8) | 1(33.3) |  |
| **Grade** |  |  |  |  |  |  |  |  |  |  |  |  |
| Low | 34(45.3) | 4(22.2) | 0(0.0) | 0.032 | 18(36.0) | 16(43.2) | 4(50.0) | 0.368 | 18(34.6) | 21(51.2) | 0(0.0) | 0.484 |
| High | 41(54.7) | 14(77.8) | 2(100,0) |  | 32(64.0) | 21(56.8) | 4(50.0) |  | 34(65.4) | 20(48.8) | 3(100.0) |  |
| **Residual tumour**  **status** |  |  |  |  |  |  |  |  |  |  |  |  |
| R0 | 49(65.3) | 10(55.6) | 2(100.0) | 0.890 | 29(58.0) | 25(67.6) | 7(87.5) | 0.221 | 32(61.5) | 28(68.3) | 2(66.7) | 0.830 |
| R1 | 24(32.0) | 8(44.4) | 0(0.0) |  | 21(42.0) | 10(27.0) | 1(12.5) |  | 20(38.5) | 11(26.8) | 1(33.3) |  |
| R2 | 2(2.7) | 2(2.7) | 0(0.0) |  | 0(0.0) | 2(5.4) | 0(0.0) |  | 0(0.0) | 2(4.9) | 0(0.0) |  |
| **Location** |  |  |  |  |  |  |  |  |  |  |  |  |
| Esophagus | - | - | - | - | - | - | - | - | - | - | - | - |
| Stomach | - | - | - |  | - | - | - |  | - | - | - | - |
| **Laurén** |  |  |  |  |  |  |  |  |  |  |  |  |
| Intestinal | 64(85.3) | 13(72.2) | 2(100.0) | 0.504 | 42(84.0) | 30(81.1) | 7(87.5) | 0.713 | 42(80.8) | 36(87.8) | 2(66.7) | 0.624 |
| Mixed | 4(5.3) | 2(11.1) | 0(0.0) |  | 4(8.0) | 2(5.4) | 0(0.0) |  | 3(5.8) | 3(7.3) | 0(0.0) |  |
| Diffuse | 7(9.3) | 3(16.7) | 0(0.0) |  | 4(8.0) | 5(13.5) | 1(12.5) |  | 7(13.5) | 2(4.9) | 1(33.3) |  |
| **MMR status** |  |  |  |  |  |  |  |  |  |  |  |  |
| pMMR | 73(97.3) | 14(77.8) | 2(100.0) | 0.019 | 50(100.0) | 33(89.2) | 6(75.0) | 0.002 | 49(94.2) | 39(95.1) | 2(66.7) | 0.428 |
| dMMR | 2(2.7) | 4(22.2) | 0(0.0) |  | 0(0.0) | 4(10.8) | 2(25.0) |  | 3(5.8) | 2(4.9) | 1(33.3) |  |
| **PD-L1 Tumour cells** |  |  |  |  |  |  |  |  |  |  |  |  |
| <1% | - | - | - | - | 46(92.0) | 23(62.2) | 6(75.0) | 0.008 | 43(84.3) | 30(75.0) | 1(33.3) | 0.041 |
| 1-49% | - | - | - |  | 3(6.0) | 14(37.8) | 1(12.5) |  | 7(13.7) | 10(25.0) | 1(33.3) |  |
| ≥50% | - | - | - |  | 1(2.0) | 0(0.0) | 1(12.5) |  | 1(2.0) | 0(0.0) | 1(33.3) |  |
| **PD-L1 Immune cells** |  |  |  |  |  |  |  |  |  |  |  |  |
| 0-10% | 46(61.3) | 3(16.7) | 1(50.0) | 0.008 | - | - | - | - | 35(68.6) | 13(32.5) | 1(33.3) | <0.001 |
| 11-50% | 23(30.7) | 14(77.8) | 0(0.0) |  | - | - | - |  | 15(29.4) | 21(52.5) | 1(33.3) |  |
| >50% | 6(8.0) | 1(5.6) | 1(50.0) |  | - | - | - |  | 1(2.0) | 6(15.0) | 1(33.3) |  |
| **PD-1 Immune cells** |  |  |  |  |  |  |  |  |  |  |  |  |
| 0-10% | 43(58.1) | 7(38.9) | 1(50.0) | 0.041 | 35(71.4) | 15(40.5) | 1(12.5) | <0.001 | - | - | - | - |
| 11-50% | 30(40.5) | 10(55.6) | 0(0.0) |  | 13(26.5) | 21(56.8) | 6(75.0) |  | - | - | - |  |
| >50% | 1(1.4) | 1(5.6) | 1(50.0) |  | 1(2.0) | 1(2.7) | 1(12.5) |  | - | - | - |  |

MMR= mismatch repair, pMMR= mismatch repair proficiency, dMMR= mismatch repair deficiency
