# Supplementary material for: Expression of PD-L1 and PD-1 in Chemoradiotherapy-Naïve Esophageal and Gastric Adenocarcinoma: Relationship With Mismatch Repair Status and Survival
Source: Front Oncol. 2019 Mar 13;9:136. doi: 10.3389/fonc.2019.00136 (PMC6425870; doi:10.3389/fonc.2019.00136)
Supplement: Supplementary file 2 [file Table_2.docx]

**Supplementary Table S2. Associations with clinicopathological factors and intercorrelation of PD-L1 and PD-1 expression in gastric cancer**

| **Factor** | **PD-L1 Tumour cells** | | | | **PD-L1 Immune cells** | | | | **PD-1 Immune cells** | | | |
| --- | --- | --- | --- | --- | --- | --- | --- | --- | --- | --- | --- | --- |
| n(%) | <1% | 1-49% | ≥50% | *P* | 0-10% | 11-50% | >50% | *P* | 0-10% | 11-50% | >50% | *P* |
| **Age** |  |  |  |  |  |  |  |  |  |  |  |  |
| mean, median  (range) | 72.2, 74.8  (42.6-94.4) | 72.8, 74.8  (42.6-94.4) | 81.7, 81.5  (77.7-86.1) | 0.184 | 70.1, 70.9  (42.6-88.8) | 76.4, 79.5  (54.5-94.4) | 83.5, 83.2  (81.5-86.1) | 0.008 | 74.1, 79.3  (42.6-88.8) | 71.6, 72.6  (48.3-94.4) | 79.2, 81.7  (73.9-83.0) | 0.343 |
| **Gender** |  |  |  |  |  |  |  |  |  |  |  |  |
| Female | 18(34.6) | 5(33.3) | 2(66.7) | 0.499 | 7(14.0) | 6(16.2) | 0(0.0) | 0.566 | 10(31.3) | 15(38.5) | 1(33.3) | 0.614 |
| Male | 34(65.4) | 10(66.7) | 1(33.3) |  | 43(86.0) | 31(83.8) | 8(100.0) |  | 22(68.8) | 24(61.5) | 2(66.7) |  |
| **T stage** |  |  |  |  |  |  |  |  |  |  |  |  |
| T1 | 6(11.5) | 2(13.3) | 0(0.0) | 0.413 | 3(6.0) | 3(8.1) | 1(12.5) | 0.038 | 2(6.3) | 6(15.4) | 0(0.0) | 0.165 |
| T2 | 10(19.2) | 4(26.7) | 1(33.3) |  | 5(10.0) | 6(16.2) | 4(50.0) |  | 7(21.9) | 8(20.5) | 2(66.7) |  |
| T3 | 22(42.3) | 7(46.7) | 2(66.7) |  | 36(72.0) | 24(64.9) | 3(37.5) |  | 14(43.8) | 17(43.6) | 1(33.3) |  |
| T4 | 14(26.9) | 2(13.3) | 0(0.0) |  | 6(12.0) | 4(10.8) | 0(0.0) |  | 9(28.1) | 8(20.5) | 0(0.0) |  |
| **N stage** |  |  |  |  |  |  |  |  |  |  |  |  |
| N0 | 23(44.2) | 8(53.3) | 1(33.3) | 0.482 | 6(12.0) | 10(27.0 | 5(62.5) | 0.001 | 12(37.5) | 22(56.4) | 1(33.3) | 0.532 |
| N1 | 9(17.3) | 3(20.0) | 1(33.3) |  | 6(12.0) | 10(27.0) | 0(0.0) |  | 8(25.0) | 3(7.7) | 1(33.3) |  |
| N2 | 9(17.3) | 2(13.3) | 1(33.3) |  | 19(38.0) | 7(18.9) | 2(25.0) |  | 5(15.6) | 8(20.5) | 0(0.0) |  |
| N3 | 11(21.2) | 2(13.3) | 0(0.0) |  | 19(38.0) | 10(27.0) | 1(12.5) |  | 7(21.9) | 6(15.4) | 1(33.3) |  |
| **M stage** |  |  |  |  |  |  |  |  |  |  |  |  |
| M0 | 42(8.8) | 14(93.3) | 3(100.0) | 0.170 | 45(90.0) | 34(91.9) | 8(100.0) | 0.407 | 26(81.3) | 35(89.7) | 2(66.7) | 0.695 |
| M1 | 10(19.2) | 1(6.7) | 0(0.0) |  | 5(10.0) | 3(8.1) | 0(0.0) |  | 6(18.8) | 4(10.3) | 1(33.3) |  |
| **Grade** |  |  |  |  |  |  |  |  |  |  |  |  |
| Low | 15(28.8) | 3(20.0) | 0(0.0) | 0.233 | 18(36.0) | 16(43.2) | 4(50.0) | 0.368 | 8(25.0) | 11(28.2) | 0(0.0) | 0.798 |
| High | 37(71.2) | 12(80.0) | 3(100.0) |  | 32(64.0) | 21(56.8) | 4(50.0) |  | 24(75.0) | 28(71.8) | 3(0.0) |  |
| **Residual tumour**  **status** |  |  |  |  |  |  |  |  |  |  |  |  |
| R0 | 35(67.3) | 12(80.0) | 2(66.7) | 0.648 | 29(58.0) | 25(67.6) | 7(87.5) | 0.221 | 23(71.9) | 28(71.8) | 2(66.7) | 0.993 |
| R1 | 12(23.1) | 1(6.7) | 1(33.3) |  | 21(42.0) | 10(27.0) | 1(12.5) |  | 6(18.8) | 7(17.9) | 1(33.3) |  |
| R2 | 5(9.6) | 2(13.3) | 0(0.0) |  | 0(0.0) | 2(5.4) | 0(0.0) |  | 3(9.4) | 4(10.3) | 0(0.0) |  |
| **Location** |  |  |  |  |  |  |  |  |  |  |  |  |
| Esophagus | - | - | - | - |  |  |  |  |  |  |  |  |
| Stomach | - | - | - |  |  |  |  |  |  |  |  |  |
| **Laurén** |  |  |  |  |  |  |  |  |  |  |  |  |
| Intestinal | 23(44.2) | 9(60.0) | 2(66.7) | 0.298 | 42(84.0) | 30(81.1) | 7(87.5) | 0.713 | 16(50.0) | 18(46.2) | 2(66.7) | 0.900 |
| Mixed | 3(5.8) | 0(0.0) | 0(0.0) |  | 4(8.0) | 2(5.4) | 0(0.0) |  | 2(6.3) | 1(2.6) | 0(0.0) |  |
| Diffuse | 26(50.0) | 6(40.0) | 1(33.3) |  | 4(8.0) | 5(13.5) | 1(12.5) |  | 14(43.8) | 20(51.3) | 1(33.3) |  |
| **MMR status** |  |  |  |  |  |  |  |  |  |  |  |  |
| pMMR | 51(98.1) | 11(73.3) | 1(33.3) | <0.001 | 50(100.0) | 33(89.2) | 6(75.0) | 0.002 | 29(90.6) | 35(89.7) | 2(66.7) | 0.459 |
| dMMR | 1(1.9) | 4(26.7) | 2(66.7) |  | 0(0.0) | 4(10.8) | 2(25.0) |  | 3(9.4) | 4(10.3) | 1(33.3) |  |
| **EBV status** |  |  |  |  |  |  |  |  |  |  |  |  |
| Negative | 52(74.3) | 13(18.6) | 2(2.9) | 0.001 | 46(65.7) | 18(25.7) | 3(4.3) | 0.068 | 31(42.5) | 36(49.3) | 3(4.1) | 0.233 |
| Positive | 0(0) | 2(2.9) | 1(1.4) |  | 1(1.4) | 1(1.4) | 1(1.4) |  | 0(0.0) | 3(4.1) | 0(0.0) |  |
| **PD-L1 Tumour cells** |  |  |  |  |  |  |  |  |  |  |  |  |
| <1% | - | - | - | - | 43(91.5) | 8(42.1) | 1(25.0) | <0.001 | 28(96.6) | 23(62.2) | 0(0.0) | <0.001 |
| 1-49% | - | - | - |  | 4(8.5) | 10(52.6) | 1(25.0) |  | 0(0.0) | 12(32.4) | 3(100.0) |  |
| ≥50% | - | - | - |  | 0(0.0) | 1(5.3) | 2(20.0) |  | 1(3.4) | 2(5.4) | 0(0.0) |  |
| **PD-L1 Immune cells** |  |  |  |  |  |  |  |  |  |  |  |  |
| 0-10% | 43(82.7) | 4(26.7) | 0(0.0) | <0.001 | - | - | - | - | 26(89.7) | 20(54.1) | 0(0.0) | 0.001 |
| 11-50% | 8(15.4) | 10(66.7) | 1(33.3) |  | - | - | - |  | 2(6.9) | 14(37.8) | 3(100.0) |  |
| >50% | 1(1.9) | 1(6.7) | 2(66.7) |  | - | - | - |  | 1(3.4) | 3(8.1) | 0(0.0) |  |
| **PD-1 Immune cells** |  |  |  |  |  |  |  |  |  |  |  |  |
| 0-10% | 28(54.9) | 0(0.0) | 1(33.3) | <0.001 | 26(56.5) | 2(10.5) | 1(25.0) | <0.001 | - | - | - | - |
| 11-50% | 23(45.1) | 12(80.0) | 2(66.7) |  | 20(43.5) | 14(73.7) | 3(75.0) |  | - | - | - |  |
| >50% | 0(0.0) | 3(20.0) | 0(0.0) |  | 0(0.0) | 3(15.8) | 0(0.0) |  | - | - | - |  |

MMR= mismatch repair, pMMR= mismatch repair proficiency, dMMR= mismatch repair deficiency, EBV = Epstein-Barr virus
