# Supplementary material for: Expression of PD-L1 and PD-1 in Chemoradiotherapy-Naïve Esophageal and Gastric Adenocarcinoma: Relationship With Mismatch Repair Status and Survival
Source: Front Oncol. 2019 Mar 13;9:136. doi: 10.3389/fonc.2019.00136 (PMC6425870; doi:10.3389/fonc.2019.00136)
Supplement: Supplementary file 3 [file Table_3.DOCX]

**Supplementary Table S3 – Associations of mismatch repair status with clinicopathological factors**

| **Factor** | **MMR status** | | |
| --- | --- | --- | --- |
|  | pMMR | dMMR | *P* |
| n(%) | 159 (91.9) | 14 (8.1) |  |
| **Age** |  |  |  |
| mean, median  (range) | 69.5, 68.9  (42.6-94.4) | 79.3, 82.5  53.4-88.8) | 0.001 |
| **Gender** |  |  |  |
| Female | 38 (23.9) | 1 (7.1) | 0.152 |
| Male | 121 (76.1) | 13 (92.9) |  |
| **T stage** |  |  |  |
| T1 | 17 (10.7) | 1 (7.1) | 0.821 |
| T2 | 29 (18.2) | 3 (21.4) |  |
| T3 | 87 (54.7) | 9 (64.3) |  |
| T4 | 26 (16.4) | 1 (7.1) |  |
| **N stage** |  |  |  |
| N0 | 49 (30.8) | 9 (64.3) | 0.012 |
| N1 | 28 (17.6) | 2 (14.3) |  |
| N2 | 39 (24.5) | 2 (14.3) |  |
| N3 | 43 (27.0) | 1 (7.1) |  |
| **M stage** |  |  |  |
| M0 | 140 (88.1) | 14 8100.0) | 0.172 |
| M1 | 19 (11.9) | 0 |  |
| **Grade** |  |  |  |
| Low | 58 (36.5) | 2 (14.3) | 0.095 |
| High | 101 (63.5) | 12 (85.7) |  |
| **Residual tumor status** |  |  |  |
| R0 | 107 (67.3) | 11 (78.6) | 0.572 |
| R1 | 44 (27.7) | 2 (14.3) |  |
| R2 | 8 (5.0) | 1 (7.1) |  |
| **Location** |  |  |  |
| Esophagus | 92 (57.9) | 6 (42.9) | 0.279 |
| Stomach | 67 (42.1) | 8 (57.1) |  |

MMR= mismatch repair, pMMR= mismatch repair proficiency,

dMMR= mismatch repair deficiency
